# ML216-Induced BLM Helicase Inhibition Sensitizes PCa Cells to the DNA-Crosslinking Agent Cisplatin

**DOI:** 10.3390/molecules27248790

**Published:** 2022-12-12

**Authors:** Xiao-Yan Ma, Jia-Fu Zhao, Yong Ruan, Wang-Ming Zhang, Lun-Qing Zhang, Zheng-Dong Cai, Hou-Qiang Xu

**Affiliations:** 1 Key Laboratory of Animal Genetics, Breeding and Reproduction in the Plateau Mountainous Region, Ministry of Education, College of Life Sciences, Guizhou University, Guiyang 550025, China; 2 Guizhou Institute of Technology, College of Food and Pharmaceutical Engineering, Guiyang 550003, China; 3 College of Animal Science, Guizhou University, Guiyang 550025, China; 4 Department of Immunology, Basic Medical College, Guizhou Medical University, Guiyang 550014, China

**Keywords:** BLM inhibitor, ML216, CDDP, combined therapy

## Abstract

Using standard DNA-damaging medicines with DNA repair inhibitors is a promising anticancer tool to achieve better therapeutic responses and reduce therapy-related side effects. Cell viability assay, neutral comet assay, western blotting (WB), and cell cycle and apoptosis analysis were used to determine the synergistic effect and mechanism of ML216, a Bloom syndrome protein (BLM) helicase inhibitor, and cisplatin (CDDP), a DNA-crosslinking agent, in PCa cells. Based on the online database research, our findings revealed that BLM was substantially expressed in PCa, which is associated with a bad prognosis for PCa patients. The combination of ML216 and CDDP improved the antiproliferative properties of three PCa cell lines. As indicated by the increased production of γH2AX and caspase-3 cleavage, ML216 significantly reduced the DNA damage-induced high expression of BLM, making PC3 more susceptible to apoptosis and DNA damage caused by CDDP. Furthermore, the combination of ML216 and CDDP increased p-Chk1 and p-Chk2 expression. The DNA damage may have triggered the ATR-Chk1 and ATM-Chk2 pathways simultaneously. Our results demonstrated that ML216 and CDDP combination therapy exhibited synergistic effects, and combination chemotherapy could be a novel anticancer tactic.

## 1. Introduction

In general, prostate cancer (PCa) has the second-highest incidence of all malignancies in the industrialized world, and in 2020, it was responsible for 6.8% of all cancer-related deaths globally [1]. External beam radiation therapy and radical prostatectomy are the mainstays of treatment for locally confined PCa [2]. Androgen deprivation therapy (ADT), which can reduce the secretion of testicular androgens and the activation of androgen receptors, is the mainstay of the newly discovered treatment for metastatic prostate cancer [3]. However, almost all patients with metastatic prostate cancer develop resistance to primary ADT, a condition known as metastatic castration-resistant prostate cancer (mCRPC). Approximately 30,000 people per year in the United States pass away from mCRPC, the most common malignancy among males [4]. Patients with mCRPC have a limited range of treatment options, the most crucial of which is chemotherapy [5]. Some organizations have investigated cases of homologous recombinant repair (HRR) disorders in mCRPC, and the results show that platinum chemotherapy has a good effect on these cases [6]. Platinum-based treatments, particularly mCRPC, have frequently demonstrated improved efficacy in cancer entities with HRR deficiency [7,8]. These treatments cause intra- and inter-strand DNA crosslinks. Additionally, CDDP for enzalutamide-resistant mCRPC is currently being evaluated in an interventional phase I research (NCT03275857). In conclusion, platinum-based therapies may become more significant for the treatment of mCRPC. CDDP is a classic cytotoxic anti-cancer drug that causes DNA damage directly by the induction of breaks or other lesions [9]. Since the FDA approved CDDP in 1978, it has been widely used as an antineoplastic drug and as a cornerstone of chemotherapy for treating many different forms of human cancers. However, its use is limited due to side effects in normal tissues and CDDP resistance [10]. Some CDDP-induced DNA damage cells cause CDDP resistance through DNA damage repair (DDR) pathways [11,12]. For CDDP-resistant cancer, DDR pathways are the key therapeutic targets.

Different kinds of cancer either activate or inactivate DDR proteins [13]. Numerous chemotherapy medicines and radiation treatments work against tumors in cancer cells by causing DNA damage. For instance, irradiation directly leads to DNA damage, and CDDP promotes nucleotide excision repair resulting in DNA crosslinks. The activation of critical factors of the DDR pathway in cancer cells is one of the leading causes of drug resistance [14]. On the other hand, cancer cells may rely more heavily on one DDR route if parts of the DDR pathways lose activity, and blocking that DDR pathway can result in synthetic lethality [15,16]. CDDP in combination with ATM or ATR inhibitors has been reported to be more effective than CDDP alone [17]. Therefore, CDDP, combined with DDR pathways’ inhibitors, is a promising candidate for molecular-targeted therapy.

A DNA helicase that unwinds complex structures of DNA such as duplexes, Holliday junctions (HJ), and G-quadruplexes (G4) is called the Bloom Syndrome Protein (BLM) helicase [18]. It belongs to the family of RecQ helicases (RECQ1, BLM, WRN, RECQ4, RECQ5) [19,20]. BLM, a critical factor in DDR pathways, helps to protect genomic integrity through HRR, telomere maintenance, and replication stress reduction [21,22,23]. Although BLM helicase inhibitors have been the subject of a few publications [24,25,26,27], ML216 is the first and only small molecule inhibitor commercially available to stop BLM activity in human cells completely. Through the competitive reduction in BLM’s DNA binding activity, potent inhibition of BLM’s DNA unwinding activity, and increased sister chromatid exchange, ML216 modifies chromosomal stability. Between 5 and 10% of alternative lengthening of telomere malignancies, like osteosarcoma, can be clinically targeted with ML216 analogs or recently created BLM inhibitors, which may offer a novel therapeutic approach for these treatment-resistant tumors [25]. Therefore, ML216 may be helpful as a potential chemotherapeutic agent in future research to target BLM inactivation more precisely. However, studies of the combination of ML216 and CDDP have not yet been reported. Therefore, we comprehensively studied the CDDP sensitization of ML216 in PCa. We wanted to determine whether the BLM inhibitor ML216 and CDDP cause DNA damage in PCa and whether they have an anti-tumor impact. According to our hypothesis, the BLM inhibitor ML216 makes PCa cells more susceptible to CDDP by obstructing DNA repair and cell cycle checkpoint functions, which leads to abnormal mitotic entry and cell death.

## 2. Results

### 2.1. BLM Could Act as a Therapeutic Drug Target for PCa

**Figure 1:**
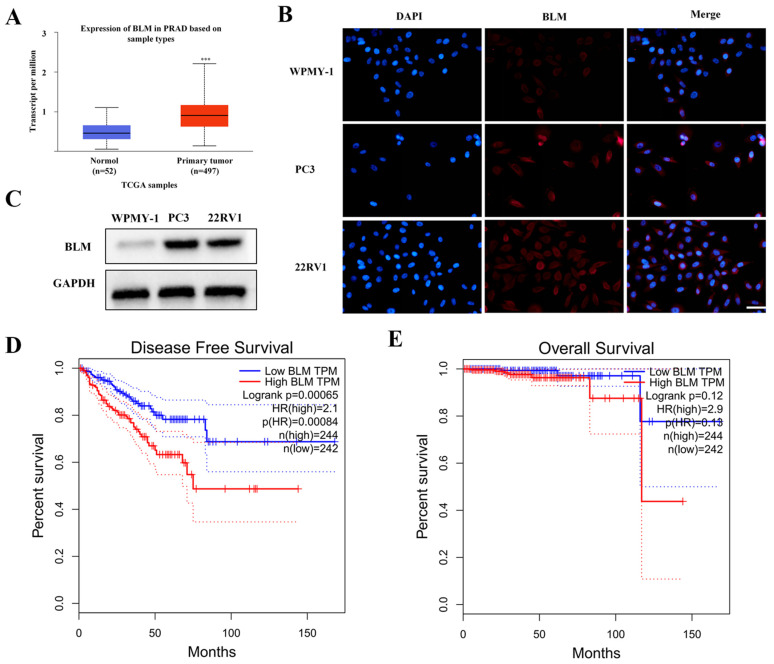
Expression of BLM in PCa. (**A**) In the TCGA database, the expression of BLM transcripts (*n* = 497) was significantly higher than that of normal tissue (*n* = 52) in PCa (*** *p* < 0.001). (**B**) The western blotting was used to analyze BLM protein expression levels in PCa cell lines. (**C**) The IF was used to analyze BLM protein levels in PCa cell lines. Scale bars: 50 μm. (**D**) The comparison of DFS between the high BLM expression group (*n* = 244) and low BLM expression group (*n* = 242) (*p* = 0.00065); *n*: the number of patients. (**E**) Comparison of overall survival in the indicated groups (*p* > 0.05).

We examined the mRNA expression of BLM genes in TCGA datasets to see whether there is a relationship between the expression of BLM genes and the emergence and progression of PCa. Figure 1A shows that BLM expression is elevated in PCa tissues (52 healthy prostate tissue samples vs. 497 PCa samples). 

As shown in Figure 1B, compared with WPMY-1 cells, BLM was overexpressed in PC3 and 22RV1 cells. We obtained similar results through IF experiments (Figure 1C). GEPIA 2.0 analysis showed that, compared with low expression of BLM, high expression was significantly lower for DFS (*p* = 0.00065) and correlated with negative prognostic features (Figure 1D,E). In conclusion, BLM is highly expressed in prostate tissues and cells, associated with a poor patient prognosis. These results suggest that BLM may be an anti-prostate cancer target.

### 2.2. ML216 Sensitizes CDDP-Induced Cytotoxicity in PCa Cells

**Figure 2:**
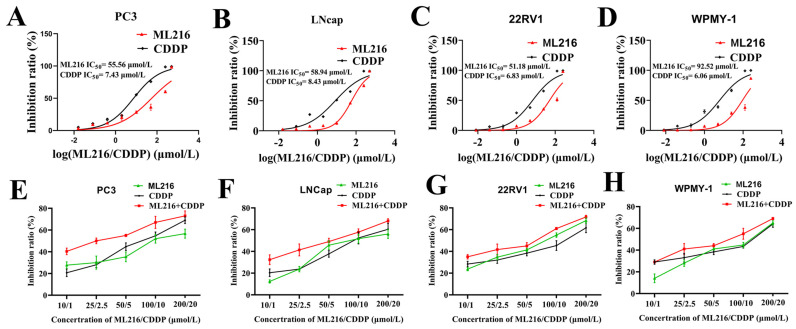
ML216 sensitizes CDDP-induced cytotoxicity in PCa cells. (**A**–**C**) Inhibition ratios in prostate cells following 48 h treatment: for PC3 cells, ML216 IC_50_−55.56 μmol/L and CDDP IC_50_−7.43 μmol/L (**A**); for LNcap cells, ML216 IC_50_−58.94 μmol/L and CDDP IC_50_−8.43 μmol/L (**B**); for 22RV1 cells, ML216 IC_50_−51.18 μmol/L and CDDP IC_50_−6.83 μmol/L (**C**); for WPMY-1 cells, ML216 IC_50_−92.52 μmol/L and CDDP IC_50_−6.06 μmol/L (**D**). (**E**–**H**) MTT assay was conducted in PC3, LNCap, 22RV1 and WPMY-1 cells treated with CDDP and ML216 for 48 h. Inhibition ratio curves were shown, respectively.

BLM, as an essential factor in the DDR pathway, can maintain genome integrity through HRR, so we hypothesize that ML216 can sensitize PCa cells to DNA crosslinkers by inhibiting BLM. Therefore, using a panel of PCa cells and human prostate cell cultures, we examined the effects of ML216 and CDDP monotherapy and a combination of both drugs. To assess the cytotoxic IC_50_ of both substances, cells were exposed to them for 48 h at various doses. As shown in Figure 2A–D, after 48 h of treatment, the IC_50_ values of ML216 and CDDP against WPMY-1, PC3, LNCap, and 22RV1 were 92.52 and 6.06 μM, 55.56 and 7.43 μM, 58.94 and 8.43 μM, and 51.18 and 6.83 μM, respectively.

To investigate whether ML216 can sensitize CDDP to the inhibitory effect of PCa cells and normal prostate cells proliferation, we first intervened with four cells (PC3, 22RV1, LNCap, WPMY-1) at different concentrations (10 μM, 25 μM, 50 μM, 100 μM, 200 μM) of ML216. In contrast, CDDP (1 μM, 2.5 μM, 5 μM, 10 μM, 20 μM) was administered to the four cell strains at different concentrations. The results revealed that compared with the ML216 group or CDDP group, the combination of ML216 and CDDP has a more potent inhibitory ability on cell proliferation in PCa cell lines (Figure 2E–H). We used CompuSyn software (ComboSyn, Inc., Version 1.0, Paramus, NJ, USA) to analyze the synergistic effects of ML216 and CDDP on the prostate cells.

In PCa cells, interactions between ML216 and CDDP were evaluated using combination index (CI) analysis. CI < 1, CI = 1, and CI > 1 represented synergistic, additive, and antagonistic effects, respectively. Table 1 shows the CI values for Fa in the range of 0.32–0.72 in prostate cells. ML216 and CDDP showed a more substantial synergistic effect in PC3 cells. When ML216 of 10 μM is combined with CDDP of 1 μM in PC3 cells, the CI value is 0.46, showing a strong synergistic effect. However, ML216 and CDDP did not show a combined effect for normal prostate cells. We selected the combination of ML216 (10 μM) and CDDP (1 μM) to study their synergistic mechanism further.

### 2.3. ML216 Enhances CDDP-Induced DSBs by Inhibiting BLM in PC3 Cells

**Figure 3:**
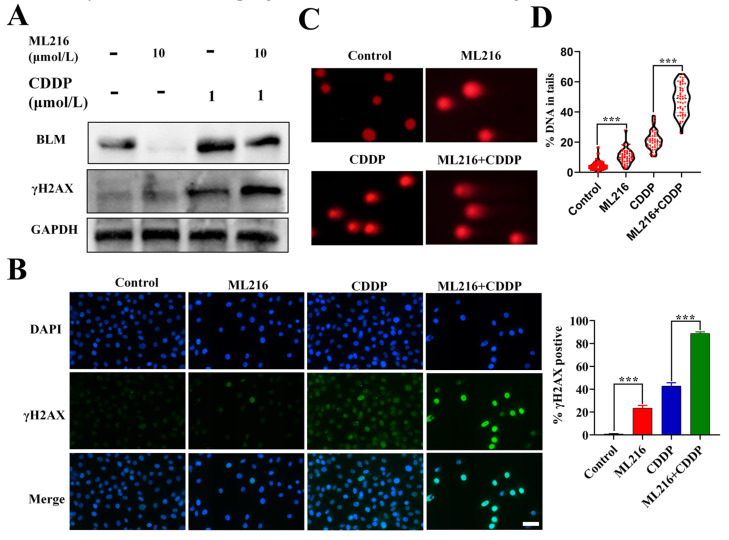
ML216 enhances CDDP-induced DSBs by inhibiting BLM in PC3 cells (**A**) PC3 cells were co-treated with CDDP (1 μM) and ML216 (10 μM) for 48 h, and WB examined BLM and γH2AX. GAPDH was used as a loading control. (**B**) Detection of γH2AX foci (green) 48 h after ML216 (10 μM) and CDDP (1 μM) treatment. Scale bars: 50 μm. (**C**) Comet assay of PC3 cells treated with ML216 and CDDP for 48 h. Scale bars: 50 μm. (**D**) Quantifying %DNA in tails in ML216 and CDDP-treated groups compared with the DMSO-treated group (control group). Quantification of 5 fields with 100 cells in total. (*** *p* < 0.001).

Based on the crucial role of BLM in positional gene stability, we hypothesized that ML216-driven BLM suppression could enhance the DNA damage of CDDP to PC3 cells. In our research, we discovered that CDDP increased the expression of BLM, whereas ML216 markedly attenuated the upregulation effect on PC3 cells (Figure 3A). 

γH2AX is an essential marker of double-strand breaks (DSBs). WB analysis of the ML216–CDDP combination’s induction of γH2AX in PC3 cells at 48 h revealed that the combination raised γH2AX expression even more, signifying more significant DSBs (Figure 3A). Similar results were obtained for PC3 cells through IF (Figure 3B). The neutral comet assay in PC3 cells was used to measure the increase in tail length caused by the ML216–CDDP combination at 48 h. The results showed that the combination further increased tail length, representing additional DSBs (Figure 3C,D). These results demonstrate that ML216 sensitizes PC3 cells to CDDP-induced DSBs by inhibiting BLM.

### 2.4. ML216 Hypersensitizes CDDP-Induced Apoptosis in PC3 Cells

**Figure 4:**
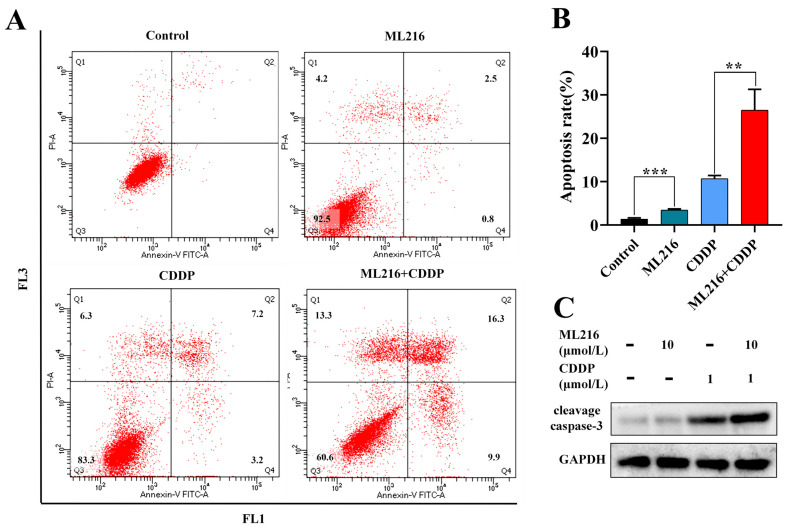
ML216 hypersensitizes CDDP-induced apoptosis in PC3 cells. (**A**) Cell apoptosis analysis on PC3 cells for control, ML216 (10 μM), CDDP (1 μM), or both treatments. (**B**) Quantitative analysis of apoptosis. (** *p* < 0.01, *** *p* < 0.001) (**C**) Protein levels of cleavage caspase 3. PC3 cells were treated with ML216, CDDP, or both for 48 h. GAPDH was used as a loading control.

Apoptosis is the process that cells start when DNA damage is blocked. We looked at how ML216 affects apoptosis by suppressing BLM because it increases CDDP-induced DSB. CDDP modestly triggered apoptosis when applied in PC3 cells at a low dose. However, ML216 (10 μM) and CDDP (1 μM) co-treatment significantly increased the apoptosis level using flow cytometry (Figure 4A). 

In control, ML216, CDDP, and ML216–CDDP-treated groups, the proportions of early (Q2) and late apoptotic (Q4) cells to total cells were 1.3, 3.4, 10.7, and 26.5%, respectively. Compared with the CDDP group, the number of apoptosis cells increased in the ML216–CDDP combination group (Figure 4B). Consistently, combined ML216 and CDDP markedly upregulated the cleavage caspase-3 compared with CDDP treatment alone (Figure 4C).

### 2.5. The Combination of ML216 and CDDP Activates ATM/Chk2 and ATR/Chk1 Signaling

**Figure 5:**
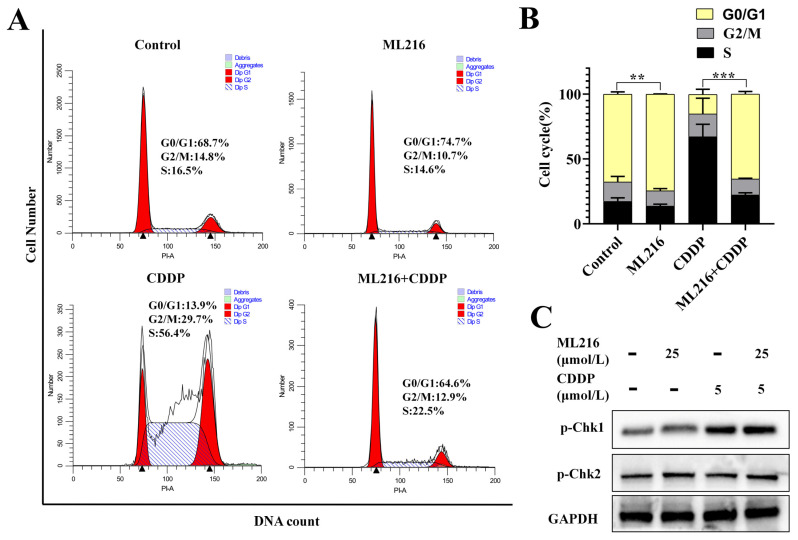
The combination of ML216 and CDDP activates ATM/Chk2 and ATR/Chk1 signaling. (**A**) Flow cytometry was used to detect the cell cycle distribution of PC3 cells after 48 h of treatment with ML216 (10 μM), CDDP (1 μM), or both. (**B**) The percentage of PC3 cells in G0/G1, S, and G2/M phases. The data were presented as mean ± SD. (** *p* < 0.01, *** *p* < 0.001) (**C**) The WB was used to analyze the protein levels of p-Chk1 and p-Chk2 in PC3 cells; GAPDH was used as a loading control.

Massive DNA damage induces the activation of cell cycle checkpoints. Therefore, we further examined the effects of ML216 on cell cycle checkpoints induced by CDDP. After CDDP was treated, PC3 cells significantly induced s-phase arrest for 48 h, indicating that S-phase checkpoints are activated to repair DNA damage. In addition, the ML216-treated group induced cell cycle arrest in the G0/G1 phase. In PC3 cells, ML216 and CDDP combination treatment induced more G0/G1-phase arrest than CDDP treatment alone and more S-phase arrest than ML216 treatment alone (Figure 5A,B).

ATM and ATR are two of the master regulators of the checkpoint pathways. The complex of ATM and the check-point kinase Chk2 responds to DSBs to induce a G0/G1 arrest, whereas ATR-ChK1 triggers S and G2 arrests. Then we examined how ML216 or CDDP affected ATM/Chk2 or ATR/Chk1 signaling pathways. As expected, the CDDP treatment group caused p-Chk1 expression upregulation, and the ML216 treatment group caused p-Chk2 expression upregulation. In combination with ML216 and CDDP, p-Chk1 and p-Chk2 expressions were both upregulated in PC3 cells (Figure 5C).

Therefore, ML216 combined with CDDP-induced activation of ATM/Chk2 and ATR/Chk1 signaling, thus leading to the activation of G0/G1 and S-phase checkpoint.

## 3. Discussion

The DNA damage response has already established itself as a crucial anticancer target, and indeed the inhibitors may increase the effectiveness of traditional cytotoxic drugs [28]. Some specific anticancer treatment methods include using DNA repair inhibitors in combination with common DNA-damaging agents to reduce the removal of toxic DNA lesions by repair [29]. Similar to platinum anticancer medications, CDDP can attach DNA by electrostatic attraction, via major or minor grooves, or via intercalation, in which case a flat section of the molecule can slide into the space between the stacked base pairs. The DNA binding process frequently results in modifications that our body’s repair system cannot undo, which triggers cell death [30,31,32]. BLM is a DNA helicase with significant functions in DDR pathways and is viewed as a promising target for cancer treatment [27]. However, studies of BLM inhibitors in combination with DNA-damaging agents are rare. In this investigation, we discovered that the combination of CDDP and the BLM inhibitor ML216 had synergistic anticancer effects on PCa. Apoptosis caused by CDDP was significantly enhanced in PCa cells by ML216. Mechanistically, ML216 combined with CDDP-induced activation of ATM/Chk2 and ATR/Chk1 signaling, which led to the activation of the G0/G1 and S-phase checkpoints, thus markedly increasing DNA damage levels.

A class of proteins that detect DSBs, chromosomal breakage, translocations, and deletions and can rectify some changes in encoding DNA repair pathways [33]. Genotoxic substances continuously and regularly assault cells. The DDR pathway employs signal sensors, transducers, and effectors to react to cellular damage. Such systems support the genome’s ability to withstand or repair the damage. DNA structural anomalies caused by DNA damage or replication stress ultimately activate the DDR mechanism [34]. The DDR pathway involves several tumor suppressor genes, including poly (ADP-ribose) polymerase (PRAP), Src, BLM, and others, each of which may help maintain genomic integrity [35]. It has long been suggested that chemosensitizing tumor cells by interfering with DNA repair is a promising anticancer tactic. Several trials have focused on this issue. Rucaparib, niraparib, and BMN673, PRAP inhibitors that target the repair system of CDDP-induced DNA damage [36], have shown promise for improving CDDP therapy, though further research is needed [37]. According to preliminary research, co-treatment with Src inhibitors such as dasatinib may re-sensitize colorectal cancers resistant to conventional platinum-based chemotherapeutic drugs, such as oxaliplatin [38]. Additionally, dasatinib has been shown to work in conjunction with doxorubicin therapy in breast cancer models and with paclitaxel therapy in ovarian cancer patients [39]. Although BLM is an essential component of DDR, it is rare to study its inhibitors in combination with DNA-damaging agents.

In this study, we examined the combined anti-tumor effect of the inhibitors of BLM and the DNA-crosslinking agent CDDP in PCa cells. The results revealed that compared with the ML216 group or CDDP group, the combination of ML216 and CDDP has a more potent inhibitory ability on cell proliferation in the PCa cell line. The combination index (CI) analysis results show that ML216 and CDDP have a strong synergistic effect in PC3 cells. Because CDDP treatment frequently results in nephrotoxicity, a combination of therapeutic methods using a minimal dose of CDDP may prevent the severe side effects without reducing the therapeutic effectiveness. In this study, we also examined the effects of ML216 and CDDP on DSBs in PC3 cells. The increase in tail length caused by the ML216-CDDP combination demonstrated that the combination further increased DSBs. These results demonstrate that ML216 is a promising anticancer candidate in combination with chemotherapy.

Cell cycle checkpoints are activated by DNA damage repair mechanisms in addition to detecting and repairing DNA damage [40]. Cell cycle checkpoints control the timing and sequencing of the cell cycle. They are essential when DNA damage or replication stress strains the genome. ATM and ATR are two of the checkpoint pathways’ master regulators. The interaction of ATM and the checkpoint kinase Chk2 results in a G1 arrest, whereas ATR-Chk1 causes S and G2 arrests. As a result, an unchecked cell cycle checkpoint can cause DNA damage to build up and encourage apoptosis [41]. Our findings demonstrated that CDDP administration caused S-phase arrest and activated the ATR/Chk1 pathway in PC3 cells. However, ML216 treatment markedly activated the ATM/Chk2 signal pathway and arrested the G0/G1 phase in PC3 cells. In addition, we discovered that ML216 and CDDP treatment induced more G0/G1-phase arrest and S-phase arrest in PC3 cells than CDDP treatment alone. Additionally, the combination of ML216 and CDDP increased the expression of p-Chk1 and p-Chk2. The DNA damage probably triggered the activation of both the ATR-Chk1 and ATM-Chk2 pathways.

## 4. Materials and Methods

### 4.1. Cell Lines

Prostate cancer cell lines PC3, 22RV1, LNCap, and human prostatic stromal myofibroblast cell line WPMY-1 were acquired from Zhong Qiao Xin Zhou Biotechnology Co., Ltd. (Shang Hai, China). In DMEM/F12 media (Gibco, Grand Island, NY, USA) with 10% FBS (04-001-1ACS; BI, Beit HaEmek, Israel) and 100 units/mL penicillin/100 g/mL streptomycin (SV30010; HyClone, Logan, UT, USA), PC3, LNCap, and WPMY-1 cells were maintained. These cells were cultivated at 37 °C in a 5% CO_2_ incubator. RPMI 1640 was used to grow 22RV1 cells (Gibco).

### 4.2. Antibodies and Reagents

The following primary antibodies were used in all cell lines: BLM (bs-12872R, Biosynthesis Biotechnology, Beijing, China); cleavage caspase 3 (ab2302, Abcam); Phospho-chk1 (Ser280) (310037, Zen BioScience, Chengdu, China); Phospho-Chk2 (Thr68) (340766, Zen BioScience); GAPDH (60004-1-Ig, Proteintech).

We purchased ML216 and CDDP from Topscience (Shanghai, China). They were suspended in DMSO at 20 mM concentration and applied in the concentration range of 0.1–500 µM.

### 4.3. Immunofluorescence (IF) Analysis

In a glass plate, cells were cultured, fixed, washed, blocked, and then incubated overnight with primary antibodies against BLM or γH2AX at 4 °C (1:100). One hour was spent incubating the fluorescent secondary antibody (1:200) at 37 °C. DAPI labeled the cell nuclei (Sigma-Aldrich, Madrid, Spain).

### 4.4. Western Blotting (WB)

PC3 cells were seeded at a density of 2 × 10^5^ cells/well into 6-well plates and allowed to grow for 24 h. After cultivation, cells were collected and lysed in RIPA buffer containing phenylmethanesulfonyl fluoride and phosStop. Insoluble cell debris was discarded following centrifugation at 13,500× *g* at 4 °C for 30 min. The supernatant containing the soluble protein was collected, and the protein concentration was measured using a bicinchoninic acid (BCA) assay. The proteins were deposited onto polyvinylidene (PDVF) membranes (Millipore Corp., Birrica, MA, USA) after electrophoresing on 10% SDS-PAGE gels. The membranes were then blocked and incubated with the primary antibody for 24 h at 4 °C. The secondary antibody was applied to the membranes after washing and developed for two hours. After an additional TBST wash, an ECL luminous reagent (Beyotime, Shanghai, China) was applied to the membranes. The internal reference was GAPDH.

### 4.5. Cell Counting Kit-8 (CCK8) Drug Sensitivity Assessment

The CCK8 technique (Saint-Bio, Shanghai, China) was used to assess the impact of ML216 or CDDP on the viability of PCa cells. PCa cells were seeded in a 96-well plate at a density of 1 × 10^4^ cells per well. The cells were treated with various doses of ML216 or CDDP after being incubated for 24 h. Following 24, 48, and 72 h of culture, the cells were treated with 10 μL of CCK8 at 37 °C for two hours. The OD_450_ level of each well was determined using the Synergy H4 automated microplate reader. The drug’s inhibition ratio and IC_50_ (50% inhibiting concentration) on cell growth were performed and calculated by OD values.

### 4.6. Cell Cycle and Apoptosis Analysis

PC3 seeded at a density of 1 × 10^5^ cells/well and developed for 24 h. Subsequently, ML216 or CDDP was added for breeding for an additional 48 h. The Annexin V/FITC Apoptosis Detection Kit (KeyGen Biotech, Jiangsu, China) was used to evaluate apoptosis after the cells were collected. The collected cells were mixed with 5 μL of FITC-Annexin V and incubated for 15 min at 25 °C in the dark. Then 5 μL of propidium iodide (PI; BD Biosciences, San Jose, CA, USA) was added and incubated for 5 min in the dark. Three independent replicates were analyzed using a flow cytometer (Canto II Plus, BD Biosciences). Cells were digested with trypsin, collected, and fixed by adding 70% ethanol at 4 °C for 24 h to conduct a cell cycle study. The cells were stained with PI for 15 min at 25 °C in the dark. A flow cytometer was used to identify the cell cycle. FlowJoV10 conducted a thorough analysis of each outcome.

### 4.7. Neutral Comet Assay

The alkaline, neutral comet assay measured DSBs in individual cells according to the instructions included in the comet assay kit (Nanjing Jian Cheng, Nanjing, China). After treatment by ML216 or CDDP for 48 h, cells were trypsinized, embedded, and lysed for 1 h. The slides were washed and incubated for 30 min. Finally, the cells were stained using PI dye for 15 min. The images quantify performed by free TriTek Comet Score software (TriTek Corp., Version 1.5., Sumerduck, VA, USA). The tail moment determines the percentage of DNA in the tail, and we analyzed at least 50 cells in each sample [42].

### 4.8. Statistical Analysis

GraphPad Prism and SPSS were used to conduct the statistical analysis. Each experiment’s data are reported as the mean ±SD, and each experiment’s data were collected from at least three separate experiments. One-way ANOVA with post hoc analysis and unpaired t-tests were used to find the statistically significant differences. Statistics were considered significant for *p* values under 0.05.

## 5. Conclusions

In conclusion, BLM plays a vital role in maintaining genomic integrity in PCa cells. The degradation of BLM via ML216 treatment sensitizes PCa cells to CDDP-mediated DNA damage. For the mechanism, ML216 combined with CDDP-induced activation of ATM/Chk2 and ATR/Chk1 signaling, thus leading to the activation of G0/G1 and S-phase checkpoint. Our results provide a rationale for further developing ML216 and CDDP combination therapy as promising therapeutic strategies for PCa.

## Figures and Tables

**Table 1:** Combination index (CI) of ML216 and CDDP in PC3, LNCap and 22RV1 cells.

ML216 (μmol/L)	CDDP (μmol/L)	PC3		LNCap		22RV1		WPMY-1	
		Fa	CI	Fa	CI	Fa	CI	Fa	CI
200	20	0.72	1.00	0.68	1.30	0.72	0.86	0.69	1.04
100	10	0.68	0.67	0.57	1.27	0.65	0.77	0.55	1.39
50	5	0.55	0.81	0.49	1.00	0.45	1.69	0.44	1.51
25	2.5	0.51	0.53	0.41	0.79	0.46	0.79	0.41	0.95
10	1	0.41	0.42	0.32	0.55	0.35	0.74	0.29	1.02

Fa: Fraction affected.

## Data Availability

All data generated or analyzed during this study are included in this article.
